# Clinical phenotypes and risk stratification for limb-threatening outcomes in acute compartment syndrome: a multicenter externally validated study

**DOI:** 10.3389/frai.2026.1936470

**Published:** 2026-09-23

**Authors:** Hong Cheng, Sitan Feng, Weiming Tan, Lei Ren, Yuanqiong Xiao, Zhenquan Liu, Jian Han, Jichong Zhu, Jianwen Cheng, Li Zhang

**Affiliations:** 1 Department of Trauma Orthopaedics and Hand Surgery, Nanxishan Hospital of Guangxi Zhuang Autonomous Region (The Second People’s Hospital of Guangxi Zhuang Autonomous Region), Guilin, China; 2 The Affiliated Hospital of Youjiang Medical University for Nationalities, Spinal, Orthopedic Surgery, Baise, China; 3 Key Laboratory of Molecular Pathology in Tumors of Guangxi Higher Education Institutions, Baise, China; 4 The First Affiliated Hospital of Guangxi Medical University, Nanning, China; 5 Guilin People’s Hospital, Guilin, China; 6 Department of Orthopedics, Ward 2, The Second Affiliated Hospital of Guilin Medical University, Guilin, China

**Keywords:** acute compartment syndrome, amputation, clinical phenotyping, risk stratification, muscle necrosis, external validation, clinical prediction

## Abstract

**Background:**

Acute compartment syndrome (ACS) is a time-sensitive limb-threatening condition that may progress to irreversible muscle necrosis and amputation. Clinical heterogeneity complicates early assessment of severe limb outcomes. This study aimed to identify clinically interpretable phenotypic patterns and evaluate whether phenotype information improves risk stratification in ACS.

**Methods:**

This multicenter retrospective cohort study included 1,475 patients with ACS from five medical centers, comprising a training cohort (*n* = 1,088) and a hospital-independent external validation cohort (*n* = 387). Data-driven clustering of 115 routinely collected clinical variables was performed without outcome information to identify exploratory phenotypes. Risk models for muscle necrosis and amputation were then developed using clinical variables alone or combined with phenotype information. Performance was evaluated by discrimination, calibration, decision-curve analysis, external validation, and predictor-contribution analysis.

**Results:**

Five exploratory clinical phenotypes were identified, reflecting distinct patterns of injury characteristics, ischemic manifestations, tissue-injury burden, and systemic compromise. In external validation, phenotype information provided little additional discrimination for muscle necrosis (ROC-AUC 0.989 for both models). For amputation, the phenotype-informed model showed higher discrimination than the base model (ROC-AUC 0.959 vs. 0.937; PR-AUC 0.763 vs. 0.681) and a lower Brier score (0.105 vs. 0.119). Risk estimates were predominantly informed by markers of muscle injury, renal function, and systemic severity.

**Conclusion:**

ACS comprises clinically heterogeneous patterns associated with different risks of severe limb outcomes. Phenotype information provided additional risk-stratification value for amputation but not for muscle necrosis. These phenotypes should be regarded as exploratory clinical patterns rather than definitive biological subtypes. Prospective validation and recalibration are required before individualized risk estimates can be considered for clinical use.

## 1 Introduction

Acute compartment syndrome (ACS) is a time-sensitive, limb-threatening orthopaedic emergency caused by elevated intracompartmental pressure and impaired tissue perfusion. Delayed recognition may lead to irreversible muscle necrosis, neurological dysfunction, infection, amputation, or death (von Keudell et al., 2015). Early identification of patients at risk of muscle necrosis or amputation is therefore essential for improving limb outcomes and guiding clinical decision-making.

Current risk assessment relies mainly on clinical signs, laboratory markers such as creatine kinase (CK), myoglobin, and renal function indices, and clinician experience (Duckworth and McQueen, 2017). However, ACS is heterogeneous: patients differ in injury mechanism, anatomical site, ischemia severity, muscle injury burden, transfer history, and systemic condition. Single symptoms, signs, or biomarkers may therefore fail to capture disease severity or distinguish subgroups with different outcome risks (Wei et al., 2023).

Clinical phenotyping offers a data-driven approach to understanding this heterogeneity (Maddali et al., 2022). Unlike traditional grouping strategies based on predefined outcomes or isolated risk factors, unsupervised machine learning can identify latent patient subgroups from candidate clinical features without using muscle necrosis or amputation outcomes. This approach may reveal descriptive injury patterns and generate hypotheses for further study, although it does not by itself establish biological subtypes.

Prediction models built on such phenotypes may help characterize risk within retrospective data (Ma et al., 2021). Muscle necrosis and amputation represent two key outcomes reflecting local tissue injury and deterioration of limb prognosis. Machine-learning models can integrate demographic data, injury characteristics, clinical signs, laboratory indicators, treatment-pathway markers, and phenotype information, but their clinical timing and intended use must be defined explicitly (Xuan et al., 2023).

Accordingly, this study aimed to develop a machine-learning framework centered on unsupervised clinical phenotyping. First, we identified latent patterns among patients with ACS using the candidate variable set described in the methods and compared their clinical characteristics and outcome rates. Second, we developed and validated supervised models for the two outcomes. Finally, we used SHAP analysis to interpret model output and evaluated archived web prototypes as research-only demonstrations.

## 2 Methods

### 2.1 Study design, population, and outcomes

This multicenter retrospective cohort study included patients with ACS treated at five participating medical centers between 2015 and 2026. After predefined eligibility criteria were applied, 1,475 patients were included: 1,088 formed the training cohort and 387 formed a hospital-independent external validation cohort. The external validation cohort was defined by hospital-level separation rather than by a temporal split or random patient-level splitting across centers, and no center contributed patients to both the training and external validation cohorts. The training cohort was subsequently divided, using the combined outcome for stratification, into a development subset (*n* = 761) and an internal-validation subset (*n* = 327). Calendar-period allocation was unavailable; therefore, temporal external validation was not assessed.

Patients were included if they were treated at a participating center between 2015 and 2026, had ACS diagnosed through clinical, laboratory, imaging, pressure-measurement, operative, or course-documentation evidence, had the required candidate clinical data available, and had documented muscle necrosis and amputation outcomes. Exclusion criteria were amputation before admission, chronic or uncertain ACS, missing key outcomes, severe missingness of core predictors, conditions substantially affecting laboratory interpretation, or duplicate admissions.

The primary outcomes were muscle necrosis and amputation. Muscle necrosis was determined by two clinicians on the basis of intraoperative findings, including muscle color, contractility, bleeding, and overall tissue viability, as documented during operative exploration or debridement. This two-clinician determination was not documented as centralized or blinded across centers; records without a definite determination were excluded as missing key outcomes. Amputation was defined as limb amputation during the index hospitalization or related treatment course because of ACS or its complications. Neither outcome was included in clustering or used as a model predictor (Collins et al., 2024).

### 2.2 Candidate predictors, preprocessing, and leakage control

Because the cohort comprised patients with established ACS, the prediction framework was intended for post-diagnostic risk stratification rather than pre-diagnostic screening. The intended prediction time point for baseline clinical and laboratory information was the initial preoperative assessment after the first available admission evaluation and laboratory panel but before the first fasciotomy or operative exploration, documentation of muscle necrosis, and amputation decision-making. Candidate predictors spanned six broad domains: baseline demographics and medical history; injury and care-pathway characteristics; initial vital signs; physical-examination findings; the first available preoperative laboratory measurements; and derived inflammatory/nutritional indices. Clearly post-treatment or post-outcome variables, including time to decompression, in-hospital transfusion, wound infection, length of stay, and the outcomes, were excluded. However, initial transfusion, glucocorticoid use, and fluid resuscitation were recorded as events occurring during hospitalization, without exact timestamps, and could have occurred before or after surgery. They are therefore treated as timing-uncertain treatment-pathway markers rather than strictly preoperative predictors. The primary analysis retained these archived variables, and all four models and *k* = 5 clustering were repeated after excluding them. Variable-specific timing, coding, preprocessing, and modeling use are reported in Supplementary Table S1, which identifies at the source-variable level whether each variable contributed to unsupervised phenotyping, supervised prediction, or both.

Preoperative CK, myoglobin, liver-function indices, and renal-function indices were retained as candidate predictors because they were clinically available at the initial assessment and reflected the patient’s current tissue-injury and systemic status. Because these biomarkers may already represent evolving muscle injury, their contribution was interpreted as risk-classification information rather than evidence of a prospective lead time before tissue injury developed.

Preprocessing parameters were fitted in the training cohort and applied unchanged to the hospital-independent external validation cohort (Riley et al., 2024). The supplied cleaned analytic matrices were complete for all retained predictors in both the training (*n* = 1,088) and external-validation (*n* = 387) cohorts (analytic missingness, 0%); therefore, the prespecified median/mode imputation rules were not activated in the analyses reported here. Accordingly, analytic missingness was 0% for all retained predictors irrespective of outcome status, and sensitivity analyses using multiple imputation or missingness indicators were not applicable to the complete analytic matrices. Source-record or pre-cleaning missingness rates could not be reconstructed from the supplied deidentified analytic files, and center-specific missingness could not be evaluated because center identifiers were unavailable. Highly skewed laboratory variables were transformed with log1p, continuous variables were standardized, and categorical variables were represented as binary or one-hot indicators. Center-specific missingness could not be evaluated because the deidentified analytic files did not retain center identifiers. Details are provided in Supplementary Tables S1, S2.

### 2.3 Unsupervised clinical phenotyping and phenotype selection

Unsupervised phenotyping was performed in the training cohort using 115 candidate variables (Chin and Collins, 2024). Outcomes and clearly post-outcome variables were excluded. Three in-hospital management indicators with unavailable patient-level timestamps (initial transfusion, glucocorticoid use, and fluid resuscitation) were present in the primary clustering matrix and could have occurred before or after surgery. Sensitivity analysis repeated *k* = 5 clustering without these variables and showed low agreement with the primary partition (ARI, 0.203), which supports cautious, exploratory interpretation of the phenotype structure.

Patient dissimilarity was calculated as the mean absolute feature-wise difference after training-fitted min–max scaling (a Gower-like distance), and clustering used a PAM-style k-medoids algorithm (Liu et al., 2024). *k* = 2–6 were compared using silhouette, Gap statistic, Davies–Bouldin and Calinski–Harabasz indices, within-medoid distance, cluster size, and clinical interpretability. For *k* = 4–6, 30 repeated 80% subsamples quantified ARI, normalized mutual information, and matched-cluster Jaccard similarity. Average-linkage hierarchical clustering and Euclidean k-medoids were used as alternative-method comparisons. *k* = 5 was retained as the primary descriptive solution based on a pragmatic balance of clinical granularity, cluster-size adequacy, and interpretability, rather than as a statistically unique or biologically validated subtype solution.

### 2.4 Phenotype interpretation, validation mapping, and outcome-risk comparison

Clusters were interpreted using dominant clinical characteristics after clustering. Outcome rates were examined only after cluster derivation. Phenotype names and risk descriptors were therefore post-hoc descriptive labels for readability; they are not causal, diagnostic, or evidence that the clusters were intrinsically outcome-defined.

The hospital-independent external validation cohort was not reclustered. Each patient was mapped to the nearest training-derived medoid using the training-fitted transformation. The confirmed hospital-level separation supports hospital-independent external validation without center overlap; however, center-stratified transportability could not be quantified because center identifiers were removed from the analytic files.

Clinical characteristics, laboratory indicators, muscle necrosis rates, and amputation rates were compared across phenotypes to determine whether unsupervised clustering identified subgroups with distinct clinical patterns and risks. Phenotype 1 served as the reference for outcome-association analyses.

### 2.5 Supervised prediction model development

Four supervised models were developed for the two outcomes using the original predictor structure. M1 and M2 were developed for muscle necrosis, whereas M3 and M4 were developed for amputation. The archived base-model matrices used 120 predictors for M1 and M3, whereas the phenotype-enhanced matrices used 125 predictors for M2 and M4. Three management indicators—initial transfusion, glucocorticoid use, and fluid resuscitation—were retained in the primary predictor set but were classified as timing-uncertain because exact patient-level timestamps were unavailable. Sensitivity analyses excluding these three variables were conducted separately.

The selected algorithm families were LightGBM for M1, CatBoost for M2, XGBoost for M3, and LightGBM for M4. Development used five-fold stratified cross-validation with random seed 42, followed by evaluation in the fixed internal-validation subset. M1 and M2 used no class weighting or imbalance-specific loss adjustment. For M3, XGBoost used the binary logistic objective with scale_pos_weight = 9.074, derived from the negative-to-positive class ratio in the fitting data. M4 used balanced class weights based on class frequencies in the fitting data. No over-sampling, under-sampling, SMOTE, or other synthetic sampling was used. The class-imbalance handling strategy was specified during model development. For the final models evaluated in external validation, data-dependent class weights were calculated from the fitting/training data only; the external-validation cohort was not used to determine class weights or sampling settings. Candidate algorithms were evaluated using prespecified conservative configurations rather than an extensive validation-informed hyperparameter search. Exact candidate configurations, final M1–M4 hyperparameters, five-fold stratified cross-validation settings, random seed, software versions, and class-imbalance handling are provided in Supplementary Table S7. Model selection considered ROC-AUC, PR-AUC, Brier score, calibration, complexity, and interpretability (Efthimiou et al., 2024). Development cross-validation, internal validation, and external validation results are reported separately.

### 2.6 Hospital-independent external validation, interpretability, and web-tool status

Final models were evaluated in the hospital-independent external validation cohort, which was not used for model fitting, hyperparameter selection, or threshold selection (Riley et al., 2024). Classification thresholds were selected separately for each model by maximizing the Youden index (sensitivity + specificity − 1) using predictions from the full training cohort. The resulting thresholds were fixed and applied unchanged to the external-validation cohort; no threshold optimization was performed using external-validation outcomes. Performance included ROC-AUC, PR-AUC, Brier score, log loss, calibration intercept and slope, calibration curves, decision-curve net benefit, sensitivity, specificity, positive and negative predictive values, F1 score, and confusion matrices. These thresholds were used for descriptive classification-performance assessment and should not be interpreted as clinically validated treatment cutoffs.

SHAP values were calculated on the independent external-validation cohort using the final fitted phenotype-enhanced models M2 and M4. Global mean absolute SHAP values and patient-level distributions describe how predictors contributed to the fitted model output. They do not estimate causal effects, treatment effects, or biological mechanisms; any clinical interpretation is therefore associational and model-specific.

Two offline HTML tools were retained as archived research prototypes corresponding to the reported model framework and demonstrating local-browser input and output flow. The tools require only the predefined clinical, examination, laboratory, and phenotype-related variables described in this study as inputs and do not require external data transmission or online server access. They have not undergone prospective workflow validation, software verification, usability testing, or regulatory evaluation and should not be considered validated medical devices or replacements for urgent clinical assessment. They must not delay fasciotomy, operative exploration, serial examination, compartment-pressure assessment, or emergency treatment. Further prospective validation, software verification, and recalibration would be required before any clinical evaluation.

### 2.7 Statistical analysis

Continuous variables are presented as medians and interquartile ranges, and categorical variables as counts and percentages. Continuous variables were compared across cohorts or phenotypes using the Kruskal-Wallis test, and categorical variables were compared using the chi-square test or Fisher exact test. Using Phenotype 1 as the reference group, odds ratios and 95% confidence intervals were calculated to evaluate associations between phenotype membership and muscle necrosis or amputation.

Model discrimination was evaluated by ROC-AUC and PR-AUC, overall accuracy by Brier score and log loss, calibration by calibration intercept, calibration slope, and calibration curves, and clinical utility by decision-curve analysis. ROC-AUC and PR-AUC confidence intervals were estimated with 500 bootstrap samples. The Youden threshold was estimated only from training predictions and was fixed before external validation. Cluster robustness used 30 repeated 80% subsamples. All analyses used random seed 42 where applicable; software details are listed in Supplementary Table S7.

## 3 Results

### 3.1 Study population and baseline characteristics

A total of 1,475 patients with ACS were included: 1,088 in the training cohort and 387 in a hospital-independent external validation cohort. Cohort allocation was performed at the hospital level, with no center overlap. The training cohort was divided into the development subset (*n* = 761) and internal-validation subset (*n* = 327). Center-level sample distributions and performance could not be reported because the deidentified analytic files did not retain center identifiers. The revised analytical workflow is shown in Figure 1.

**Figure 1:**
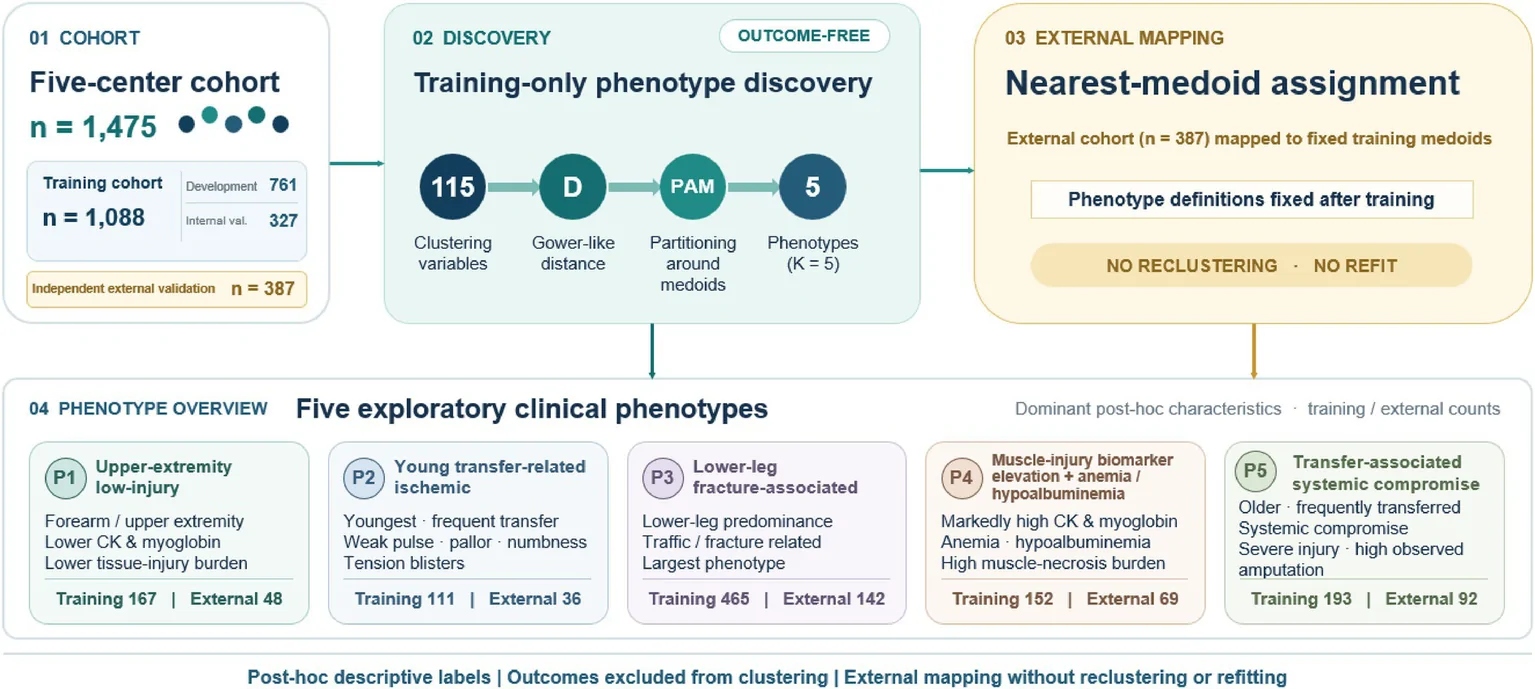
Study design and analytical workflow. The figure summarizes cohort construction and dataset partitioning; data preprocessing, unsupervised phenotyping, validation-cohort phenotype mapping, phenotype characterization, supervised model development, external validation, SHAP interpretation, and web-based deployment; and the structure of the four prediction models. M1 and M2 were developed for muscle necrosis prediction, whereas M3 and M4 were developed for amputation prediction. M1 and M3 used the candidate clinical and laboratory predictor set, whereas M2 and M4 additionally incorporated phenotype information.

In the full cohort, 640 patients developed muscle necrosis (43.4%) and 150 underwent amputation (10.2%). Muscle-necrosis rates in development, internal validation, and external validation were 42.3, 42.2, and 46.5%; corresponding amputation rates were 9.9, 10.1, and 10.9%. These distributions were broadly comparable but do not address center-level heterogeneity.

Baseline characteristics were broadly similar across the three analytic subsets. The supplied cleaned analytic matrices contained zero missing cells; consequently, the prespecified imputation rules were not activated. Center-stratified comparisons were not possible because center identifiers were absent. Detailed characteristics and variable-level timing are shown in Table 1 and Supplementary Tables S1–S3.

**Table 1:** Baseline characteristics of the study cohorts.

Variable	Development subset (*n* = 761)	Internal-validation subset (*n* = 327)	External validation cohort (*n* = 387)	*p* value
Age, years	40.00 [27.00, 52.00]	40.00 [27.00, 50.50]	40.00 [26.00, 53.00]	0.885
BMI	24.22 [22.76, 25.71]	24.09 [22.99, 25.35]	24.35 [22.93, 26.13]	0.280
Hemoglobin, g/L	99.0 [80.0, 127.0]	101.0 [81.0, 132.0]	97.0 [76.0, 128.5]	0.310
White blood cell count, 10^9/L	12.79 [8.97, 17.40]	13.38 [8.79, 18.00]	13.52 [9.43, 18.57]	0.298
Neutrophil count	10.13 [6.72, 15.21]	10.30 [6.58, 15.71]	10.45 [6.67, 15.83]	0.867
Albumin, g/L	35.60 [27.40, 40.50]	36.00 [26.90, 41.15]	34.04 [24.90, 40.45]	0.162
Creatinine, μmol/L	79.0 [64.0, 109.0]	81.0 [66.0, 121.0]	84.2 [63.3, 131.6]	0.497
Creatine kinase, U/L	3,452 [1,008, 10,689]	3,452 [1,008, 10,856]	3,478 [1,071, 11,873]	0.348
Myoglobin	152.0 [52.0, 617.0]	152.0 [48.1, 897.0]	152 [51, 1,056]	0.570
PT, s	12.30 [11.30, 14.50]	13.40 [11.50, 14.90]	13.00 [11.40, 14.90]	0.045
Male sex	638 (83.8)	287 (87.8)	329 (85.0)	
Injury cause				0.618
Traffic accident	237 (31.1)	93 (28.4)	119 (30.7)	
Ground-level fall	110 (14.5)	35 (10.7)	48 (12.4)	
Crush injury	84 (11.0)	43 (13.1)	33 (8.5)	
Other	330 (43.4)	156 (47.7)	187 (48.3)	
Anatomical site				0.662
Forearm	162 (21.3)	65 (19.9)	75 (19.4)	
Lower leg	489 (64.3)	209 (63.9)	253 (65.4)	
Other sites	110 (14.5)	53 (16.2)	59 (15.2)	
Interhospital transfer	421 (55.3)	179 (54.7)	211 (54.5)	0.963
Fracture at compartment site	504 (66.2)	211 (64.5)	270 (69.8)	0.300
Pain on passive stretch	240 (31.5)	97 (29.7)	110 (28.4)	0.533
Weak or absent arterial pulse	400 (52.6)	166 (50.8)	216 (55.8)	0.378
Skin pallor	496 (65.2)	196 (59.9)	225 (58.1)	0.043
Distal numbness	623 (81.9)	264 (80.7)	294 (76.0)	0.058
Tension blister	297 (39.0)	139 (42.5)	144 (37.2)	0.343
Shock	111 (14.6)	48 (14.7)	59 (15.2)	0.955
Intraoperative muscle necrosis	322 (42.3)	138 (42.2)	180 (46.5)	0.353
Amputation	75 (9.9)	33 (10.1)	42 (10.9)	0.868

This table summarizes the baseline demographic, injury-related, laboratory, clinical, and outcome characteristics of the development subset, internal-validation subset, and independent external validation cohort. Continuous variables are presented as median [interquartile range], and categorical variables are presented as *n* (%). Group comparisons were performed using the Kruskal–Wallis test for continuous variables and the chi-square test or Fisher’s exact test for categorical variables, as appropriate.

### 3.2 Identification of unsupervised clinical phenotypes

The primary *k* = 5 solution had a silhouette coefficient of 0.070, Davies–Bouldin index of 3.769, Gap statistic of 0.796, and cluster sizes of 111–465. In 30 repeated 80% subsamples, the median ARI was 0.766 (interquartile range, 0.673–0.863), median normalized mutual information was 0.717, and median matched-cluster Jaccard similarity was 0.762. *k* = 4 and *k* = 6 had median ARIs of 0.724 and 0.793, respectively. Thus, *k* = 5 was not uniquely superior in terms of resampling stability. It was retained as the primary descriptive solution because it provided a pragmatic balance between clinical granularity, adequate cluster sizes, and interpretability, rather than because it represented a statistically unique or maximally stable partition.

The cluster solution was method- and predictor-set dependent. Agreement with average-linkage hierarchical clustering was negligible (ARI, 0.003), agreement with Euclidean k-medoids was low (ARI, 0.293), and repeating *k* = 5 clustering after excluding initial transfusion, glucocorticoid use, and fluid resuscitation yielded ARI 0.203. Two-dimensional projection also showed substantial overlap. These findings support interpretation of the five groups as exploratory clinical patterns rather than sharply separated or biologically fixed subtypes (Figure 2; Supplementary Figure S1 and Supplementary Table S4).

**Figure 2:**
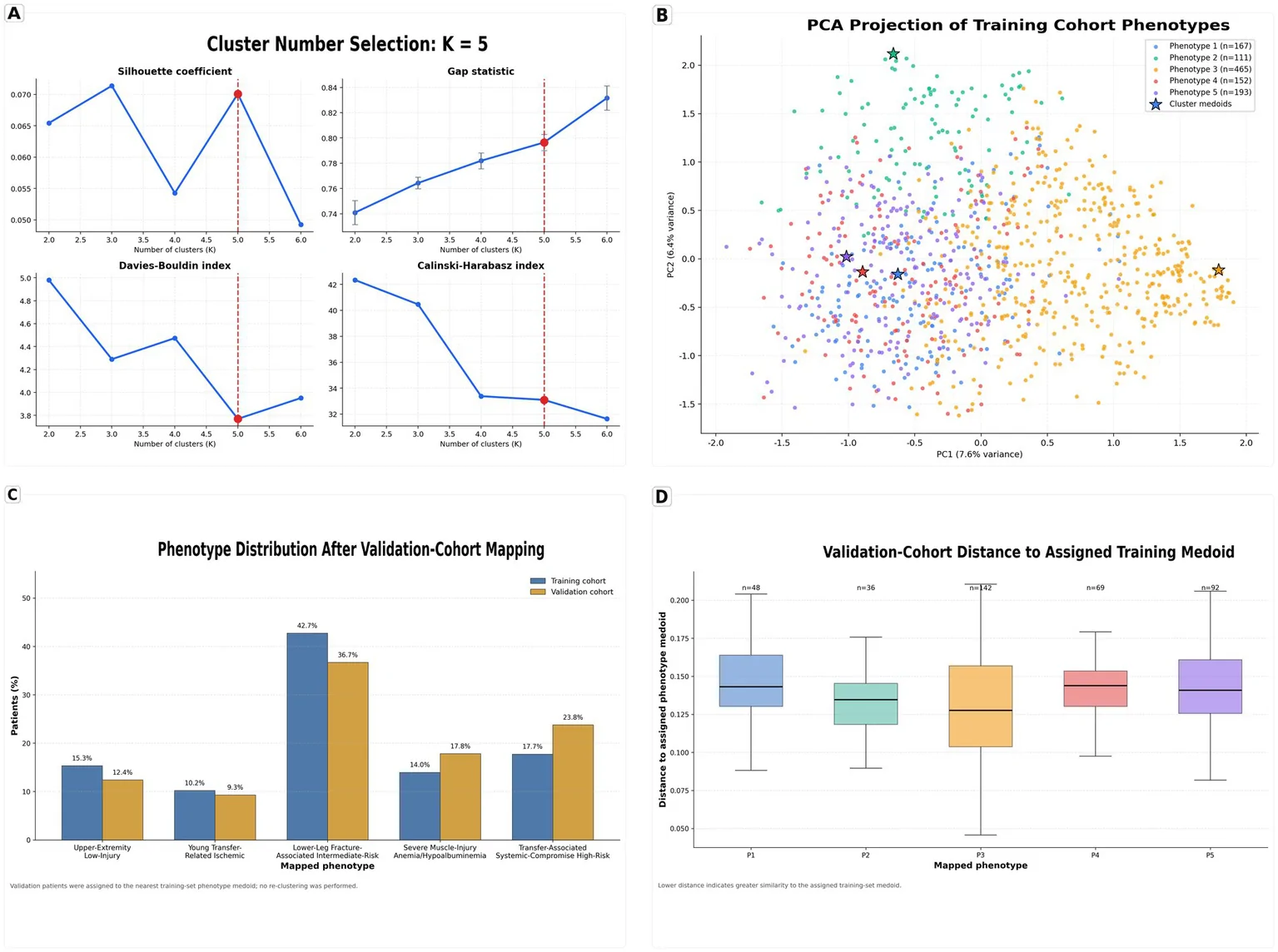
Unsupervised phenotyping and validation mapping. **(A)** Cluster-number selection based on clustering performance metrics across candidate *k* values. **(B)** Two-dimensional visualization of training-cohort patients according to the final unsupervised phenotype assignment. **(C)** Distribution of the five phenotypes in the training and independent validation cohorts. **(D)** Validation-cohort phenotype mapping based on proximity to training-derived phenotype prototypes or medoids. The validation cohort was not reclustered, thereby preserving its role as an independent validation dataset.

### 3.3 Phenotype mapping in the hospital-independent external validation cohort

The 387 patients from hospitals independent of the training cohort were mapped to the nearest training-derived medoid without reclustering. Phenotypes 1–5 included 48, 36, 142, 69, and 92 patients, respectively. This demonstrates operational assignment in a hospital-independent external cohort, although center-specific transportability estimates were unavailable.

Median nearest-medoid distances were approximately 0.128–0.144. Because no validated distance threshold for out-of-distribution detection was prespecified, these values are reported descriptively and are not labeled as proof of stable external assignment.

### 3.4 Clinical profiles and outcome risks across phenotypes

The five exploratory patterns showed descriptive differences. P1 was characterized by upper-extremity involvement and lower tissue-injury markers; P2 by younger age, transfer, and ischemic signs; P3 by lower-leg fracture-associated injuries; P4 by severe muscle injury with anemia and hypoalbuminemia; and P5 by transfer-associated systemic compromise. These labels were assigned after clustering and should not be interpreted as diagnoses or causal risk categories.

Observed outcome rates varied across patterns. P4 had muscle-necrosis rates of 65.1% in training and 59.4% in external validation; P5 had amputation rates of 17.6 and 19.6%. These post-hoc gradients help describe the clusters but were not used to derive them and do not overcome the low silhouette or method dependence.

The profiles and post-hoc outcome rates are shown in Figure 3 and Table 2. Detailed characteristics and unadjusted phenotype–outcome associations are provided in Supplementary Tables S5–S6. All phenotype–outcome language is descriptive rather than causal.

**Figure 3:**
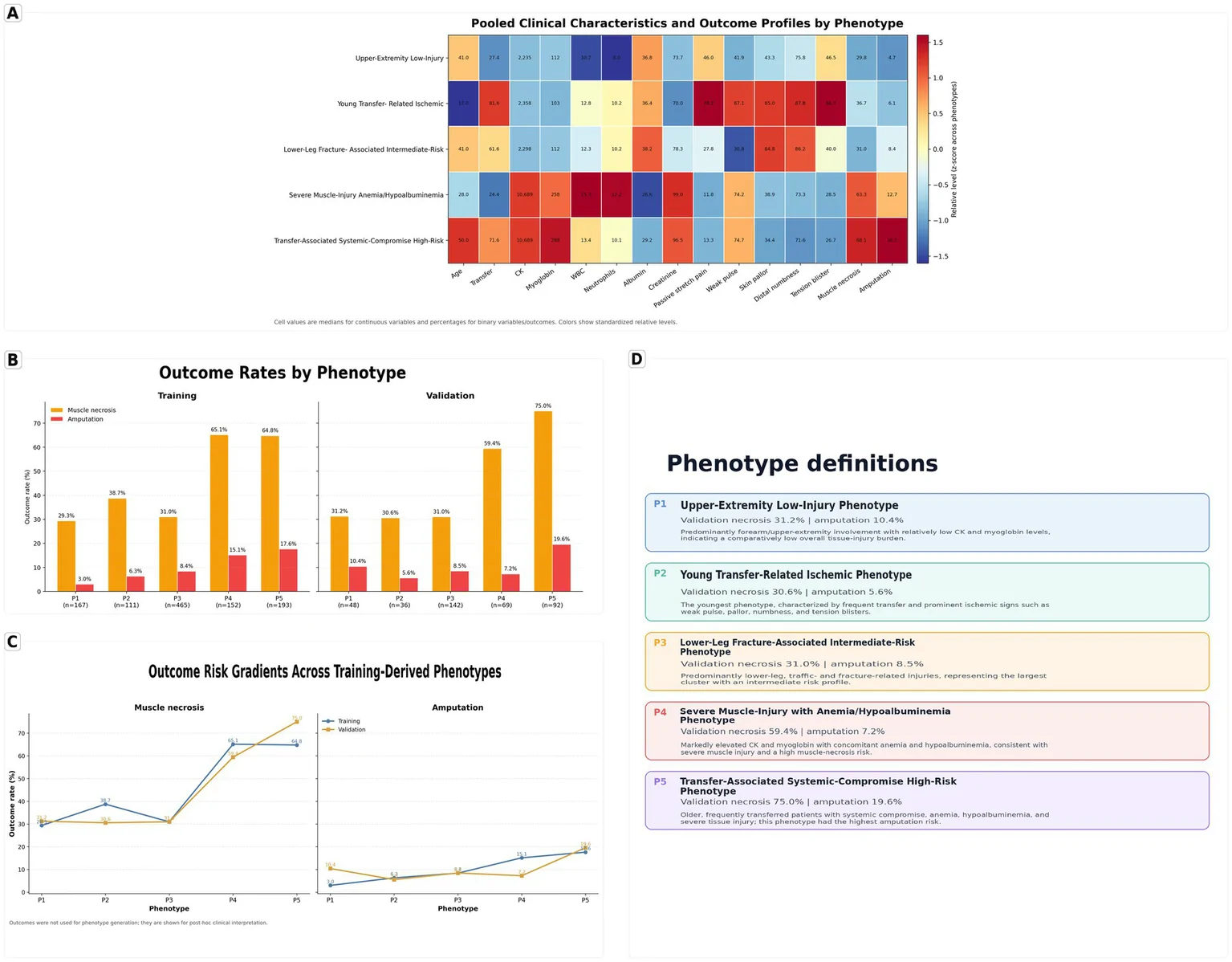
Clinical profiles and outcome risks across phenotypes. **(A)** Heatmap showing the clinical and laboratory profiles of the five unsupervised phenotypes. **(B)** Phenotype-specific outcome rates in the training and validation cohorts. **(C)** Outcome-risk gradients across phenotypes. **(D)** Clinical definitions of the five phenotypes based on their dominant features and observed outcome patterns. The identified phenotypes were named as follows: P1, upper-extremity low-injury phenotype; P2, young transfer-related ischemic phenotype; P3, lower-leg fracture-associated phenotype; P4, severe muscle-injury with anemia/hypoalbuminemia phenotype; and P5, transfer-associated systemic-compromise phenotype.

**Table 2:** Clinical definitions and outcome risks of the five phenotypes.

Phenotype	Phenotype name	Training *n* (%)	External validation *n* (%)	Dominant characteristics	Training muscle necrosis, %	Training amputation, %	Validation muscle necrosis, %	Validation amputation, %
Phenotype 1	Upper-extremity low-injury phenotype	167 (15.3)	48 (12.4)	Predominantly forearm/upper-extremity involvement with relatively low CK and myoglobin levels, indicating a comparatively low overall tissue-injury burden.	29.3	3.0	31.2	10.4
Phenotype 2	Young transfer-related ischemic phenotype	111 (10.2)	36 (9.3)	The youngest phenotype, characterized by frequent transfer and prominent ischemic signs such as weak pulse, pallor, numbness, and tension blisters.	38.7	6.3	30.6	5.6
Phenotype 3	Lower-leg fracture-associated phenotype	465 (42.7)	142 (36.7)	Predominantly lower-leg, traffic- and fracture-related injuries, representing the largest cluster with predominantly lower-leg and fracture-associated injuries.	31.0	8.4	31.0	8.5
Phenotype 4	Severe muscle-injury with anemia/hypoalbuminemia phenotype	152 (14.0)	69 (17.8)	Markedly elevated CK and myoglobin with concomitant anemia and hypoalbuminemia, consistent with severe muscle injury and a higher observed muscle-necrosis rate.	65.1	15.1	59.4	7.2
Phenotype 5	Transfer-associated systemic-compromise phenotype	193 (17.7)	92 (23.8)	Older, frequently transferred patients with systemic compromise, anemia, hypoalbuminemia, and severe tissue injury; this phenotype showed a high observed amputation rate.	64.8	17.6	75.0	19.6

This table presents the final clinical names, dominant characteristics, sample sizes, and observed outcome rates of the five unsupervised phenotypes in the training and validation cohorts. Phenotype labels were assigned post hoc according to dominant clinical and laboratory characteristics for descriptive purposes. Outcomes were not used during phenotype derivation. Observed outcome rates are presented descriptively and should not be interpreted as defining the clusters or establishing causal risk categories.

### 3.5 Supervised model development and internal validation

M1 and M3 used 120 predictors, whereas M2 and M4 used 125 predictors including phenotype information. Five-fold development ROC-AUCs were 0.983, 0.981, 0.906, and 0.906 for M1–M4, respectively; corresponding PR-AUCs were 0.981, 0.968, 0.529, and 0.544. In internal validation, M1–M4 achieved ROC-AUCs of 0.986, 0.989, 0.936, and 0.937, PR-AUCs of 0.975, 0.985, 0.669, and 0.649, and Brier scores of 0.042, 0.039, 0.104, and 0.094, respectively.

Phenotype enhancement produced little difference in discrimination for muscle necrosis. For amputation, M4 showed similar internal discrimination to M3 but a lower Brier score, supporting further evaluation of its incremental value in external validation.

### 3.6 Hospital-independent external validation of M1–M4

For muscle necrosis, M1 achieved ROC-AUC 0.989 (95% CI, 0.980–0.995), PR-AUC 0.986 (95% CI, 0.974–0.995), and Brier score 0.042; M2 achieved ROC-AUC 0.989 (95% CI, 0.980–0.995), PR-AUC 0.986 (95% CI, 0.974–0.995), and Brier score 0.041. These nearly identical results indicate little incremental discrimination from phenotype information for muscle necrosis.

For amputation, M3 achieved ROC-AUC 0.937 (95% CI, 0.910–0.962), PR-AUC 0.681 (95% CI, 0.557–0.800), and Brier score 0.119; M4 achieved ROC-AUC 0.959 (95% CI, 0.938–0.978), PR-AUC 0.763 (95% CI, 0.641–0.878), and Brier score 0.105. M4 also showed higher specificity (0.881 vs. 0.783), PPV (0.481 vs. 0.342), and F1 score (0.628 vs. 0.500), although sensitivity was slightly lower (0.905 vs. 0.929). These findings indicate incremental external-validation performance from phenotype information for amputation, but do not by themselves establish clinical benefit or transportability.

Model performance is summarized in Figure 4 and Table 3, with calibration and threshold details in Supplementary Tables S8−S10. These are hospital-independent external validation results based on nonoverlapping centers; center-specific performance could not be estimated because center identifiers were unavailable in the analysis files. After exclusion of initial transfusion, glucocorticoid use, and fluid resuscitation, external-validation ROC-AUCs were 0.989, 0.987, 0.934, and 0.953 for M1–M4, respectively, indicating only modest changes in model discrimination.

**Figure 4:**
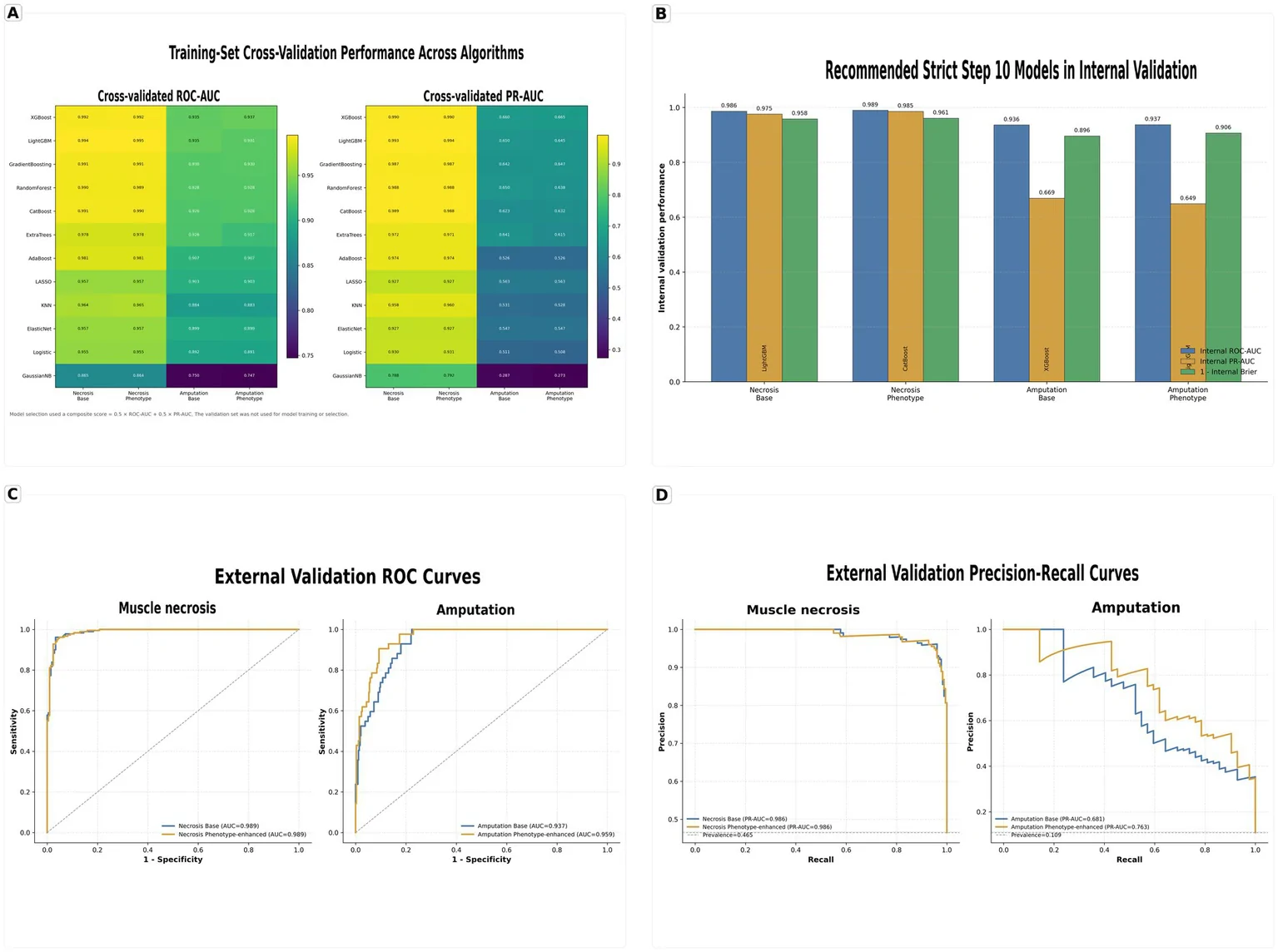
Model development and external validation of M1–M4 prediction models. **(A)** Candidate algorithm screening during supervised model development. **(B)** Internal-validation performance of the recommended models selected from the development workflow. **(C)** External validation ROC curves for muscle necrosis and amputation prediction. **(D)** External validation precision-recall curves for muscle necrosis and amputation prediction. M1 and M2 were used for muscle necrosis prediction, whereas M3 and M4 were used for amputation prediction. M2 and M4 incorporated phenotype information in addition to the candidate clinical and laboratory predictor set.

**Table 3:** Internal and external validation performance of M1–M4.

Model	Outcome	Predictor set	Algorithm	Number of predictors	Development CV ROC-AUC, mean ± SD	Development CV PR-AUC, mean ± SD	Internal ROC-AUC	Internal PR-AUC	Internal Brier score	External ROC-AUC (95% CI)	External PR-AUC (95% CI)	External Brier score	Sensitivity	Specificity	PPV	NPV	F1 score
M1	Muscle necrosis	Base	LightGBM	120	0.983 ± 0.012	0.981 ± 0.011	0.986	0.975	0.042	0.989 (0.980–0.995)	0.986 (0.974–0.995)	0.042	0.961	0.961	0.956	0.966	0.958
M2	Muscle necrosis	Phenotype-enhanced	CatBoost	125	0.981 ± 0.010	0.968 ± 0.025	0.989	0.985	0.039	0.989 (0.980–0.995)	0.986 (0.974–0.995)	0.041	0.950	0.961	0.955	0.957	0.953
M3	Amputation	Base	XGBoost	120	0.906 ± 0.036	0.529 ± 0.115	0.936	0.669	0.104	0.937 (0.910–0.962)	0.681 (0.557–0.800)	0.119	0.929	0.783	0.342	0.989	0.500
M4	Amputation	Phenotype-enhanced	LightGBM	125	0.906 ± 0.033	0.544 ± 0.123	0.937	0.649	0.094	0.959 (0.938–0.978)	0.763 (0.641–0.878)	0.105	0.905	0.881	0.481	0.987	0.628

This table summarizes the performance of the four final supervised prediction models. M1 and M2 were developed for muscle necrosis prediction, and M3 and M4 were developed for amputation prediction. M1 and M3 used the candidate predictor set without phenotype information, whereas M2 and M4 additionally included phenotype information. Model performance was evaluated using development cross-validation, internal validation, and independent external validation. Reported metrics include ROC-AUC, PR-AUC, Brier score, sensitivity, specificity, positive predictive value, negative predictive value, and F1 score.

### 3.7 Calibration and decision curve analysis

External-validation calibration curves showed imperfect agreement between predicted and observed risks (Figure 5A). M1 and M2 showed broadly similar calibration patterns for muscle necrosis, whereas the amputation models showed greater deviation from ideal calibration. Probability estimates should therefore be interpreted cautiously and require prospective recalibration before individualized risk communication.

In decision-curve analysis, model net benefit exceeded both treat-all and treat-none across continuous threshold ranges of 0.04–0.80 for M1, 0.03–0.80 for M2 (Figure 5C), 0.04–0.27 for M3, and 0.04–0.29 for M4 (Figure 5D). Isolated advantages at very high amputation thresholds were not emphasized because only 42 amputation events were available in external validation. Figure 5B is restricted to the clinically interpretable probability range.

**Figure 5:**
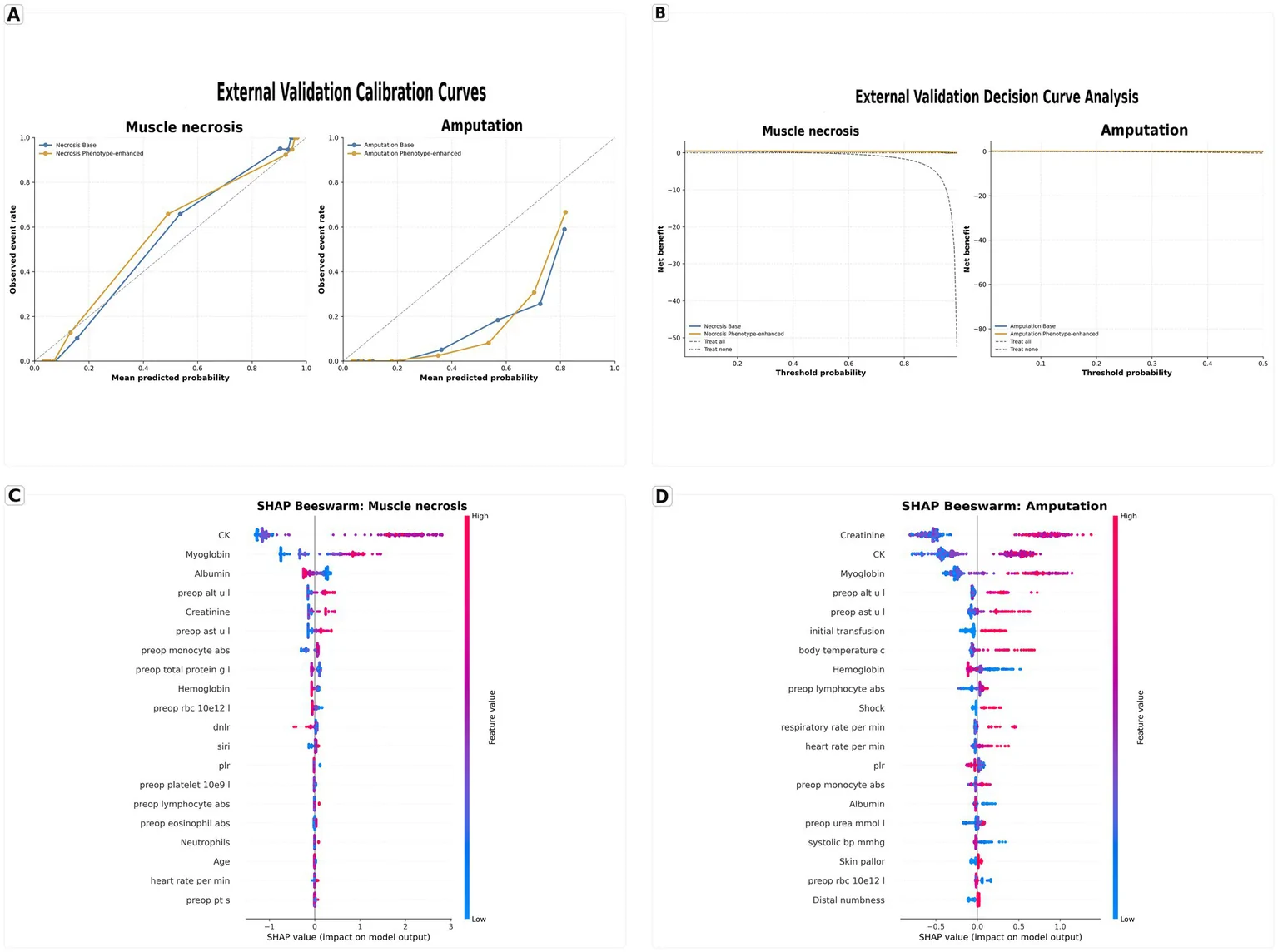
Calibration, clinical utility, and SHAP interpretation. **(A)** External validation calibration curves of the final prediction models. **(B)** Decision curve analysis evaluating the clinical net benefit of the models across threshold probabilities. **(C)** SHAP beeswarm plot generated using the independent external-validation cohort for the phenotype-enhanced muscle necrosis model. **(D)** SHAP beeswarm plot for the phenotype-enhanced amputation model. In the SHAP plots, each dot represents one patient; the horizontal axis indicates the SHAP value, and color represents the feature value. Positive SHAP values indicate contributions toward higher model output, whereas negative SHAP values indicate contributions toward lower model output.

### 3.8 SHAP-based model interpretation

For M2, the largest mean absolute SHAP contributions came from CK, myoglobin, ALT, AST, total protein, hemoglobin, creatinine, monocyte count, and albumin. These values describe contributions to model output rather than causal effects. The prominent contributions of CK, myoglobin, liver enzymes, and renal-function indices indicate that the model was strongly informed by the patient’s existing biochemical tissue-injury burden, including markers that may reflect already-evolving muscle injury.

For M4, prominent SHAP contributors included creatinine, myoglobin, CK, AST, ALT, hemoglobin, temperature, initial transfusion, heart rate, and PLR. Initial transfusion was recorded during hospitalization and could have occurred before or after surgery; it is therefore interpreted as a timing-uncertain treatment-pathway and severity marker, not as a strictly preoperative or causal determinant.

Phenotype indicators did not dominate the SHAP rankings. Together with the low silhouette and method dependence, this supports viewing phenotyping as an exploratory representation of heterogeneity rather than a substitute for original clinical predictors or an established diagnostic taxonomy.

### 3.9 Web-tool deployment

The two offline HTML interfaces are retained as archived research prototypes demonstrating phenotype assignment and M1–M4 output flow. The prototypes run locally without uploading data but have not undergone prospective workflow validation, software verification, usability testing, or regulatory evaluation. They are not approved medical devices and must not be used to delay serial examination, compartment-pressure assessment, fasciotomy, operative exploration, or other emergency treatment. Further prospective validation, software verification, and recalibration would be required before any clinical evaluation.

## 4 Discussion

This study presents an exploratory phenotyping and prediction framework for ACS (Loftus et al., 2022). The revised analysis confirms that the data contain clinically interpretable patterns but also shows low geometric separation, method dependence, and sensitivity of the cluster partition to three timing-uncertain in-hospital management variables. The five phenotypes should therefore be viewed as descriptive hypotheses for future validation.

Phenotype enhancement did not materially improve muscle-necrosis discrimination but showed incremental external-validation performance for amputation (Topol, 2019). SHAP analysis indicated that model outputs were primarily influenced by muscle-injury and systemic-severity markers; however, these associations represent model-specific feature contributions rather than causal determinants of necrosis or amputation risk.

The contribution of unsupervised learning in this study is hypothesis generation: it organizes heterogeneous candidate clinical data into interpretable patterns without using outcomes during clustering (Seymour et al., 2019). The low silhouette, timing-variable sensitivity, and limited alternative-method agreement mean that these patterns should not be described as definitive biological subtypes (Pan et al., 2025).

P4 and P5 displayed higher observed adverse-outcome rates, but those rates were assigned and examined after clustering (Pan et al., 2025). The descriptive labels do not establish that anemia, transfer, or other features causally produced the outcomes; treatment timing, care pathways, and documentation may also contribute (Wei et al., 2023; Zhang et al., 2020).

The very high discrimination of the muscle-necrosis models requires cautious interpretation. CK, myoglobin, liver enzymes, and renal-function indices were among the dominant contributors in the SHAP analysis (Regmi et al., 2026; Lim, 2025). These biomarkers are clinically relevant indicators of tissue-injury burden, but they may already reflect substantial or evolving muscle injury at the time of the first preoperative blood draw rather than precede the underlying injury process. Therefore, the high discrimination of M1 and M2 likely reflects, at least in part, recognition of the patient’s existing biochemical injury state associated with subsequent operative confirmation of muscle necrosis. Accordingly, the present retrospective data do not demonstrate that M1 or M2 can predict future incident muscle necrosis before tissue injury develops or with a defined lead time sufficient to alter management. The models are more appropriately interpreted as post-diagnostic retrospective risk-classification models that identify patients with a high likelihood of subsequently documented operative muscle necrosis based on their available clinical and biochemical status. Prospective studies with strictly time-stamped predictors and a prespecified prediction horizon before operative confirmation will be required to determine whether true early prediction is achievable.

For amputation, M4 improved external-validation discrimination and threshold-based specificity relative to M3, but internal discrimination was not higher and both models were substantially miscalibrated. Phenotype enhancement may capture interactions relevant to a complex outcome (Lin et al., 2024), yet its clinical value remains uncertain pending recalibration and prospective validation in additional independent settings (Pan et al., 2025). Because amputation was an uncommon outcome in this cohort, positive predictive values remained influenced by outcome prevalence despite strong discrimination. Therefore, PPV should be interpreted together with sensitivity, specificity, calibration, and decision-curve results rather than as an independent indicator of clinical certainty.

SHAP values offer model-specific explanations, not causal estimates. Because several laboratory biomarkers reflecting tissue injury burden and systemic response are biologically correlated, SHAP rankings should be interpreted as model-dependent attribution patterns rather than independent effects of individual predictors. Creatinine, shock, transfusion, hemoglobin, and other variables may act as severity (Brien et al., 2026) or treatment-pathway proxies (Salvo et al., 2024); their SHAP direction should not be interpreted as evidence that modifying the variable would change outcome risk. The overlap between SHAP rankings and phenotype profiles indicates descriptive consistency within this dataset. It does not independently validate the cluster solution or establish mechanistic coherence (Wu et al., 2023).

In a potential future clinical workflow, these models should be considered adjunctive risk-stratification tools rather than autonomous decision-making systems. Given that the current cohort consisted of patients with established ACS, the models are most appropriately considered after initial emergency assessment and availability of routinely collected clinical and laboratory information. Emergency physicians, trauma teams, or orthopaedic surgeons may use the predicted risk estimates to support identification of patients who may require closer monitoring, expedited reassessment, or multidisciplinary discussion. However, decisions regarding fasciotomy, operative exploration, compartment-pressure assessment, and other emergency interventions should remain based on urgent clinical evaluation and established diagnostic criteria (Vasey et al., 2022).

Strengths include the relatively large ACS cohort, evaluation of two severe outcomes, a fixed development/internal/external-validation structure with hospital-level separation, explicit cluster resampling and alternative-method comparisons, timing-sensitivity analyses, SHAP-based assessment of injury-related predictors, and comprehensive discrimination, calibration, decision-curve, and explanation reporting (Vasey et al., 2022).

The analysis separates development, internal validation, and a hospital-independent external validation cohort and reports discrimination, calibration, and threshold-based performance. The hospital-level separation and absence of center overlap were confirmed by the authors. Nevertheless, deidentification removed center identifiers, preventing center-specific distributions, heterogeneity analyses, and site-level performance estimates.

Limitations are substantial. First, the study is retrospective and susceptible to selection, documentation, and treatment-pathway bias. Second, although external validation used hospital-level separation without center overlap, center identifiers were unavailable in the deidentified analytic files, preventing center-level outcome rates, center-specific missingness, heterogeneity analyses, and site-level performance estimates. Third, muscle necrosis was determined by two clinicians from intraoperative findings, but adjudication was not documented as centralized or blinded. Fourth, exact timestamps for three in-hospital management variables were unavailable and the variables could have occurred before or after surgery; excluding them materially changed the *k* = 5 partition (ARI, 0.203) and modestly reduced prediction performance. Fifth, the *k* = 5 solution had low silhouette and poor agreement with alternative clustering methods. Sixth, the strong muscle-necrosis discrimination may partly reflect the prominent contribution of biomarkers representing already-evolving tissue injury, and the retrospective data did not permit estimation of a clinically actionable prediction horizon before operative confirmation of necrosis. Seventh, calibration—especially for amputation—was inadequate, so recalibration is required before probability communication. Eighth, the archived HTML tools have not undergone software verification, usability testing, prospective validation, or decision-impact evaluation. The models and tools must not replace urgent clinical judgment or treatment.

## 5 Conclusion

This study identifies five exploratory, clinically interpretable ACS patterns and provides hospital-independent external validation performance for four prediction models. Phenotype enhancement retained incremental discrimination for amputation but not muscle necrosis. Low cluster separation, method and timing-variable sensitivity, the prominent contribution of injury-related biomarkers, absent center-stratified analyses, and miscalibration preclude clinical deployment. Additional prospective temporal and geographically diverse validation with standardized outcome adjudication and recalibration is required.

## Data Availability

The raw data supporting the conclusions of this article will be made available by the authors, without undue reservation.

## Supplementary material

The Supplementary material for this article can be found online at:

https://www.frontiersin.org/articles/10.3389/frai.2026.1936470/full#supplementary-material

SUPPLEMENTARY FIGURE S1 Additional unsupervised phenotyping results. **(A)** Sample size distribution of the five training-derived phenotypes. **(B)** Outcome rates across phenotypes. **(C)** Expanded clinical profile heatmap of the unsupervised phenotypes. **(D)** Unadjusted phenotype-outcome association analysis using Phenotype 1 as the reference. These supplementary analyses provide additional support for the clinical heterogeneity and outcome-risk gradients captured by the unsupervised phenotype structure. AUC, area under the curve; CK, creatine kinase; DCA, decision curve analysis; IQR, interquartile range; PAM, partitioning around medoids; PPV, positive predictive value; PR-AUC, area under the precision-recall curve; ROC-AUC, area under the receiver operating characteristic curve; SHAP, Shapley additive explanations.

SUPPLEMENTARY FIGURE S2 Phenotype naming and validation mapping details. **(A)** Phenotype profile heatmap used to support clinical phenotype naming. **(B)** Proposed clinical phenotype definition cards summarizing the dominant characteristics of each phenotype. **(C)** Outcome rates by mapped phenotype in the validation cohort. **(D)** Statistical heterogeneity across phenotype-level clinical characteristics. These results support the clinical interpretability and external mapping feasibility of the training-derived phenotypes. AUC, area under the curve; CK, creatine kinase; DCA, decision curve analysis; IQR, interquartile range; PAM, partitioning around medoids; PPV, positive predictive value; PR-AUC, area under the precision-recall curve; ROC-AUC, area under the receiver operating characteristic curve; SHAP, Shapley additive explanations.

SUPPLEMENTARY FIGURE S3 Candidate algorithm screening and cross-validation results. **(A)** Cross-validation performance heatmap of candidate algorithms across prediction tasks. **(B)** Best-performing candidate models by task during development. **(C)** Comparison between base and phenotype-enhanced models during cross-validation. **(D)** Ranking of candidate algorithms according to composite performance. These analyses were used to guide model selection before strict internal validation. AUC, area under the curve; CK, creatine kinase; DCA, decision curve analysis; IQR, interquartile range; PAM, partitioning around medoids; PPV, positive predictive value; PR-AUC, area under the precision-recall curve; ROC-AUC, area under the receiver operating characteristic curve; SHAP, Shapley additive explanations.

SUPPLEMENTARY FIGURE S4 Strict development/internal-validation model performance. **(A)** Internal-validation Brier score heatmap across candidate algorithms and prediction tasks. **(B)** Internal-validation performance of the recommended models. **(C)** Comparison between base and phenotype-enhanced models in strict internal validation. **(D)** External validation performance summary of the final models. These results summarize model selection after the training cohort was divided into development and internal-validation subsets. AUC, area under the curve; CK, creatine kinase; DCA, decision curve analysis; IQR, interquartile range; PAM, partitioning around medoids; PPV, positive predictive value; PR-AUC, area under the precision-recall curve; ROC-AUC, area under the receiver operating characteristic curve; SHAP, Shapley additive explanations.

SUPPLEMENTARY FIGURE S5 Additional external-validation results. **(A)** Confusion matrices of the final models in the independent validation cohort. **(B)** Summary of external validation performance metrics. **(C)** External validation ROC curves. **(D)** External validation precision-recall curves. Threshold-based metrics were calculated using thresholds derived from the training cohort and applied unchanged to the external validation cohort. AUC, area under the curve; CK, creatine kinase; DCA, decision curve analysis; IQR, interquartile range; PAM, partitioning around medoids; PPV, positive predictive value; PR-AUC, area under the precision-recall curve; ROC-AUC, area under the receiver operating characteristic curve; SHAP, Shapley additive explanations.

SUPPLEMENTARY FIGURE S6 SHAP interpretation of the phenotype-enhanced muscle necrosis model based on the independent external-validation cohort. SHAP values represent model-specific feature contributions rather than causal effects. **(A)** SHAP beeswarm plot for the phenotype-enhanced muscle necrosis model. **(B)** SHAP feature-importance bar plot showing the mean absolute SHAP value of top predictors. **(C)** Directional SHAP contribution of phenotype variables. **(D)** Targeted SHAP contribution plot of clinically relevant variables. The model was mainly driven by markers of muscle injury burden, nutritional status, renal function, and hematologic status. AUC, area under the curve; CK, creatine kinase; DCA, decision curve analysis; IQR, interquartile range; PAM, partitioning around medoids; PPV, positive predictive value; PR-AUC, area under the precision-recall curve; ROC-AUC, area under the receiver operating characteristic curve; SHAP, Shapley additive explanations.

SUPPLEMENTARY FIGURE S7 SHAP interpretation of the phenotype-enhanced amputation model based on the independent external-validation cohort. SHAP values represent model-specific feature contributions rather than causal effects. **(A)** SHAP beeswarm plot for the phenotype-enhanced amputation model. **(B)** SHAP feature-importance bar plot showing the mean absolute SHAP value of top predictors. **(C)** Directional SHAP contribution of phenotype variables. **(D)** Targeted SHAP contribution plot of clinically relevant variables. The model was mainly driven by renal function, muscle injury burden, systemic compromise, transfusion-related severity, anemia, and shock-related variables. AUC, area under the curve; CK, creatine kinase; DCA, decision curve analysis; IQR, interquartile range; PAM, partitioning around medoids; PPV, positive predictive value; PR-AUC, area under the precision-recall curve; ROC-AUC, area under the receiver operating characteristic curve; SHAP, Shapley additive explanations.

SUPPLEMENTARY FIGURE S8 Web calculator and offline deployment. **(A)** Prototype offline web interface for phenotype assignment. **(B)** Offline M1–M4 prediction calculator for individualized risk estimation. **(C)** Example output showing phenotype assignment, predicted risk probabilities, and risk categories. **(D)** Deployment workflow of the local browser-based calculator. The web tools were developed for research demonstration and risk communication, and they are not intended to replace clinical judgment or urgent surgical decision-making. AUC, area under the curve; CK, creatine kinase; DCA, decision curve analysis; IQR, interquartile range; PAM, partitioning around medoids; PPV, positive predictive value; PR-AUC, area under the precision-recall curve; ROC-AUC, area under the receiver operating characteristic curve; SHAP, Shapley additive explanations.

SUPPLEMENTARY TABLE S1 Variable dictionary, coding, preprocessing, timing classification, and modeling use. This table lists all candidate variables, including variable names, coding rules, preprocessing procedures, modeling use, and timing relative to the intended prediction point. Initial transfusion, glucocorticoid use, and fluid resuscitation are classified as timing-uncertain in-hospital management variables because exact patient-level timestamps were unavailable and these variables could have occurred before or after surgery. AUC, area under the curve; CK, creatine kinase; DCA, decision curve analysis; IQR, interquartile range; PAM, partitioning around medoids; PPV, positive predictive value; PR-AUC, area under the precision-recall curve; ROC-AUC, area under the receiver operating characteristic curve; SHAP, Shapley additive explanations.

SUPPLEMENTARY TABLE S2 Preprocessing and leakage-control summary. This table summarizes data preprocessing steps, including duplicate removal, missing-value imputation, log transformation, standardization, categorical encoding, exclusion of outcome or post-treatment variables, and prevention of information leakage. All preprocessing parameters were estimated from the training data and then applied to validation data. AUC, area under the curve; CK, creatine kinase; DCA, decision curve analysis; IQR, interquartile range; PAM, partitioning around medoids; PPV, positive predictive value; PR-AUC, area under the precision-recall curve; ROC-AUC, area under the receiver operating characteristic curve; SHAP, Shapley additive explanations.

SUPPLEMENTARY TABLE S3 Full baseline characteristics across study cohorts. This table provides the complete baseline comparison of all available candidate variables across the development subset, internal-validation subset, and independent external validation cohort. Continuous variables are presented as median [interquartile range], and categorical variables are presented as *n* (%) or dominant category distributions, as appropriate. AUC, area under the curve; CK, creatine kinase; DCA, decision curve analysis; IQR, interquartile range; PAM, partitioning around medoids; PPV, positive predictive value; PR-AUC, area under the precision-recall curve; ROC-AUC, area under the receiver operating characteristic curve; SHAP, Shapley additive explanations.

SUPPLEMENTARY TABLE S4 Cluster-number evaluation metrics. This table reports clustering evaluation metrics for candidate cluster numbers from *k* = 2 to *k* = 6, including silhouette coefficient, Davies–Bouldin index, Calinski–Harabasz index, Gap statistic, and cluster-size considerations. The final number of phenotypes was selected by integrating statistical performance, cluster stability, sample-size adequacy, and clinical interpretability. AUC, area under the curve; CK, creatine kinase; DCA, decision curve analysis; IQR, interquartile range; PAM, partitioning around medoids; PPV, positive predictive value; PR-AUC, area under the precision-recall curve; ROC-AUC, area under the receiver operating characteristic curve; SHAP, Shapley additive explanations.

SUPPLEMENTARY TABLE S5 Detailed clinical characteristics of the five phenotypes. This table presents detailed demographic, injury-related, laboratory, clinical-sign, and outcome characteristics across the five unsupervised phenotypes. The table supports the clinical interpretation and naming of the phenotypes. AUC, area under the curve; CK, creatine kinase; DCA, decision curve analysis; IQR, interquartile range; PAM, partitioning around medoids; PPV, positive predictive value; PR-AUC, area under the precision-recall curve; ROC-AUC, area under the receiver operating characteristic curve; SHAP, Shapley additive explanations.

SUPPLEMENTARY TABLE S6 Phenotype-outcome association analysis. This table reports the association between phenotype membership and adverse outcomes, including muscle necrosis and amputation, using Phenotype 1 as the reference group. The analysis evaluates whether the unsupervised phenotypes capture clinically meaningful outcome-risk gradients. AUC, area under the curve; CK, creatine kinase; DCA, decision curve analysis; IQR, interquartile range; PAM, partitioning around medoids; PPV, positive predictive value; PR-AUC, area under the precision-recall curve; ROC-AUC, area under the receiver operating characteristic curve; SHAP, Shapley additive explanations.

SUPPLEMENTARY TABLE S7 Hyperparameter and algorithm settings. This table summarizes the candidate algorithms, model settings, conservative hyperparameter search space, class-imbalance handling strategies, and model-selection criteria used during supervised machine-learning development. AUC, area under the curve; CK, creatine kinase; DCA, decision curve analysis; IQR, interquartile range; PAM, partitioning around medoids; PPV, positive predictive value; PR-AUC, area under the precision-recall curve; ROC-AUC, area under the receiver operating characteristic curve; SHAP, Shapley additive explanations.

SUPPLEMENTARY TABLE S8 All algorithm results in development and internal validation. This table provides the full performance results of all candidate algorithms across the four prediction tasks. Metrics include cross-validation performance in the development subset and performance in the internal-validation subset. AUC, area under the curve; CK, creatine kinase; DCA, decision curve analysis; IQR, interquartile range; PAM, partitioning around medoids; PPV, positive predictive value; PR-AUC, area under the precision-recall curve; ROC-AUC, area under the receiver operating characteristic curve; SHAP, Shapley additive explanations.

SUPPLEMENTARY TABLE S9 Complete external validation performance of M1–M4. This table provides the full external validation performance of the final M1–M4 models in the independent validation cohort, including discrimination, calibration-related metrics, and classification metrics. AUC, area under the curve; CK, creatine kinase; DCA, decision curve analysis; IQR, interquartile range; PAM, partitioning around medoids; PPV, positive predictive value; PR-AUC, area under the precision-recall curve; ROC-AUC, area under the receiver operating characteristic curve; SHAP, Shapley additive explanations.

SUPPLEMENTARY TABLE S10 Thresholds and confusion matrices. This table reports the training-derived classification thresholds and corresponding confusion-matrix metrics of the final models in the independent validation cohort, including true positives, false positives, true negatives, false negatives, sensitivity, specificity, positive predictive value, negative predictive value, and F1 score. AUC, area under the curve; CK, creatine kinase; DCA, decision curve analysis; IQR, interquartile range; PAM, partitioning around medoids; PPV, positive predictive value; PR-AUC, area under the precision-recall curve; ROC-AUC, area under the receiver operating characteristic curve; SHAP, Shapley additive explanations.
