# Supplementary material for: Clinical phenotypes and risk stratification for limb-threatening outcomes in acute compartment syndrome: a multicenter externally validated study
Source: Front Artif Intell. 2026 Sep 23;9:1936470. doi: 10.3389/frai.2026.1936470 (PMC13642743; doi:10.3389/frai.2026.1936470)
Supplement: Supplementary file 1 [file Data_Sheet_1.ZIP › Supplementary Material 3.pdf]

广西医科大学第一附属医院
THE FIRST AFFILIATED HOSPITAL OF
GUANGXI MEDICAL UNIVERSITY

FIRST AFFILIATED HOSPITAL of GUANGXI MEDICAL UNIVERSITY
ETHICAL REVIEW COMMITTEE Approval Notice

Approval Number: 2026-E0568

Title: Clinical Phenotyping and Machine Learning-Based Risk Prediction of Limb-Threatening Outcomes in Compartment Syndrome

Research Contents: This is a multicenter retrospective study of patients diagnosed with acute compartment syndrome between 2015 and 2026. The study will collect existing clinical data from participating medical centers, including demographic information, medical history, injury characteristics, admission vital signs, laboratory results, inflammatory and nutritional indicators, early clinical symptoms and signs, treatment-related information, and clinical outcomes. The primary outcomes are muscle necrosis and amputation.

The study will use anonymized retrospective data only. No additional intervention, examination, treatment, biological sample collection, or follow-up visit will be performed. Patients with chronic compartment syndrome, unclear diagnosis, missing key outcomes, severe missing core variables, or repeated admissions will be excluded according to predefined criteria.

The analysis will include data cleaning, variable coding, missing-value handling, and statistical analysis. Unsupervised machine-learning methods will be used to identify clinically meaningful phenotypes based on early clinical variables. Supervised machine-learning models will then be developed and validated to predict muscle necrosis and amputation. Model performance will be evaluated using discrimination, calibration, decision curve analysis, and classification metrics. SHAP analysis will be used to interpret model predictions. All results will be reported in aggregate form without disclosing identifiable patient information.

Applicant: Weiming Tan

Application Department: Pathology department

Date of Application: June 21, 2026

Date of Approval: June 23, 2026

Conclusion: This paper fully considered and protected the rights and interests of the study objects. It meets the criteria of Ethical Review Committee. The Medical Ethics Committee of First Affiliated Hospital of Guangxi Medical University has approved the protocol.

Signature:

(Vice) Director of Ethical Review Committee

First Affiliated Hospital of Guangxi Medical University

Date: June 23, 2026
