# Supplementary material for: Clinical phenotypes and risk stratification for limb-threatening outcomes in acute compartment syndrome: a multicenter externally validated study
Source: Front Artif Intell. 2026 Sep 23;9:1936470. doi: 10.3389/frai.2026.1936470 (PMC13642743; doi:10.3389/frai.2026.1936470)
Supplement: Supplementary file 1 [file Data_Sheet_1.ZIP › Supplementary Material 1.html]

Compartment Syndrome Phenotype Classifier


# Compartment Syndrome Clinical Phenotype Classifier

Browser-based phenotype assignment derived from the training-defined unsupervised learning phenotypes.
This simplified web model estimates the closest clinical phenotype and displays phenotype-level risks for muscle necrosis and amputation.

## Patient input

Age, years

Anatomical siteLower legForearmOther sites

Injury causeTraffic accidentGround-level fallCrush injuryOther injury causes

Interhospital transferNoYes

Creatine kinase, U/L

Myoglobin

Hemoglobin, g/L

Albumin, g/L

Creatinine, μmol/L

White blood cell count, 10^9/L

Neutrophil count, 10^9/L

Pain on passive stretchNoYes

Weak or absent arterial pulseNoYes

Skin pallorNoYes

Distal numbnessNoYes

Tension blisterNoYes

ShockNoYes

Assign phenotype
Load high-risk example
Reset

Important: this is a simplified prototype-distance web model for phenotype assignment and visual demonstration.
It uses selected clinical variables and phenotype prototypes; the full research model used a larger processed feature set.

## Phenotype output

Enter patient information and click **Assign phenotype**.

## Phenotype definitions


Phenotype-level risks are descriptive rates observed after phenotype mapping; outcomes were not used during phenotype assignment.
