# Supplementary material for: Clinical phenotypes and risk stratification for limb-threatening outcomes in acute compartment syndrome: a multicenter externally validated study
Source: Front Artif Intell. 2026 Sep 23;9:1936470. doi: 10.3389/frai.2026.1936470 (PMC13642743; doi:10.3389/frai.2026.1936470)
Supplement: Supplementary file 1 [file Data_Sheet_1.ZIP › Supplementary Material 2.html]

Compartment Syndrome M1-M4 Offline Prediction Models


# Compartment Syndrome M1–M4 Offline Prediction Models

Self-contained offline calculator for four final supervised models:
M1 muscle necrosis base model, M2 muscle necrosis phenotype-enhanced model,
M3 amputation base model, and M4 amputation phenotype-enhanced model.
The browser applies training-set preprocessing parameters and evaluates exported tree models locally.

## Patient input

Values left blank will be replaced by the training-set median or mode. For phenotype-enhanced models M2/M4, select the phenotype assigned by the unsupervised phenotype tool.

Phenotype for M2/M4


Use the Step 13 phenotype classifier or manually select the nearest phenotype.

Predict M1–M4
Auto-compute derived indices
Load training medians/modes
Load high-risk example

Research-use prototype. This offline calculator is not a substitute for clinical assessment, urgent surgical evaluation, or institutional decision-making.

## Prediction output

Fill in patient information and click **Predict M1–M4**.

## Model description


Thresholds are training-derived Youden thresholds from the strict development/internal-validation workflow.
The independent validation set was not used to derive thresholds.
